# *Dendroceruslui* (Hymenoptera, Ceraphronoidea, Megaspilidae): a new species of *Dendroceruscarpenteri* species-group from China

**DOI:** 10.3897/BDJ.11.e108742

**Published:** 2023-10-09

**Authors:** Fang Li, Shanshan Cui, Yixin Huang, Xuan Wang, Huayan Chen, Xu Wang

**Affiliations:** 1 Anhui Provincial Key Laboratory of the Conservation and Exploitation of Biological Resources, Anhui Provincial Key Laboratory of Molecular Enzymology and Mechanism of Major Diseases, College of Life Sciences, Anhui Normal University, Wuhu, China Anhui Provincial Key Laboratory of the Conservation and Exploitation of Biological Resources, Anhui Provincial Key Laboratory of Molecular Enzymology and Mechanism of Major Diseases, College of Life Sciences, Anhui Normal University Wuhu China; 2 Collaborative Innovation Center of Recovery and Reconstruction of Degraded Ecosystem in Wanjiang Basin Co-founded by Anhui Province and Ministry of Education, School of Ecology and Environment, Wuhu, China Collaborative Innovation Center of Recovery and Reconstruction of Degraded Ecosystem in Wanjiang Basin Co-founded by Anhui Province and Ministry of Education, School of Ecology and Environment Wuhu China; 3 Key Laboratory of Zoological Systematics and Evolution, Institute of Zoology, Chinese Academy of Sciences, Beijing, China Key Laboratory of Zoological Systematics and Evolution, Institute of Zoology, Chinese Academy of Sciences Beijing China; 4 Key Laboratory of Plant Resources Conservation and Sustainable Utilization, South China Botanical Garden, Chinese Academy of Sciences, Guangzhou, China Key Laboratory of Plant Resources Conservation and Sustainable Utilization, South China Botanical Garden, Chinese Academy of Sciences Guangzhou China

**Keywords:** hyperparasitoid, taxonomy, morphology, 28S rDNA

## Abstract

**Background:**

One new species of the genus *Dendrocerus* Ratzeburg, 1852, *D.lui* Li and Wang **sp. nov.** is described. A key to Chinese species of males is provided. The 28S sequence was generated to supplement the association of both sexes of the new species.

**New information:**

One new species of the genus *Dendrocerus* Ratzeburg, 1852, *D.lui* Li and Wang **sp. nov.** is described.

## Introduction

Megaspilidae belongs to the superfamily Ceraphronoidea (Hymenoptera) and contains 13 genera worldwide (Johnson and Musetti 2004, Bijoy and Rajmohana 2014). The genus *Dendrocerus* Ratzeburg, 1852 is distinctive in Megaspilidae because the agriculturally relevant species *Dendroceruscarpenteri* (Curtis, 1829) serves as a model species to study the behaviour and ecology of parasitic wasps (Curtis 1829, Ratzeburg 1852, Burks et al. 2016, Trietsch et al. 2018). *Dendrocerus* species are usually primary parasitic wasps of Neuroptera and Diptera or hyperparasitic wasps of Hemiptera and Coleoptera, especially as hyperparasitoids of aphids (Aphididae) (Mikó et al. 2011, Bijoy and Rajmohana 2014). Species of *Dendrocerus* occur in all habitats, except for the Polar Regions, with 120 described species worldwide (Mikó et al. 2011). Forty-eight species of *Dendrocerus* have been recorded from the Oriental and Palaearctic Regions (Johnson and Musetti 2004, Trietsch et al. 2018) and seven species are known from China: *D.angustus* Dessart, 1999, *D.carpenteri*, *D.aphidum* Rondani, 1877, *D.laticeps* Hedicke, 1929, *D.laevis* Ratzeburg, 1852, *D.anisodontus* Wang, Chen & Mikó, 2021 and *D.bellus* Wang, Chen & Mikó, 2021 (Ratzeburg 1852, Rondani 1877, Hedicke 1929, Chen and Huang 1989, Wang et al. 2021).

Based on the shape of male antennae, *Dendrocerus* was divided into five species-groups, i.e. *halidayi*, *carpenteri*, *serricornis*, *punctipes* and *penmaricus* (*Dessart 1995, Bijoy and Rajmohana 2014, Pezzini and Köhler 2020*). The male antennal flagella of the *D.carpenteri* species-group are serrated in several segments and these saw-winged flagellomeres are triangular or vaguely trapezoidal in outline in length over the width (Dessart 1995).

In the present paper, we describe one new species of *Dendrocerus*, *D.lui* Li and Wang, sp. nov. under the *D.carpenteri* species-group, bringing the species number of this genus to eight from China.

## Materials and methods

Specimens were obtained from sweep-net and yellow-pan traps. Specimens are deposited in the Insect Collection of Auhui Normal University (AHNU), Wuhu, China.

Species of *Dendrocerus* were determined using the characters of Wang et al. (2021). The dry specimens were mounted on a pointcard. Colour images of dried specimens were taken with a Leica M205A stereomicroscope equipped with a Leica DFC-500 digital camera. Adobe Photoshop Version 2020 software was used to correct the brightness and sharpness of the images before typesetting.

Male genitalia were obtained by the following steps. First, the metasoma was soaked in 35% hydrogen peroxide (H_2_O_2_) for 20 minutes, then washed with distilled water for 30 minutes and then dehydrated with 25–50% ethanol for 15 minutes. Finally, the metasoma was soaked in glycerol for dissection placed in glycerine (Trietsch et al. 2017).

Abbreviations, morphological terms (Table 1) and Genitalia terminology follows Miko and Deans (2009). Measurements are given in microns.

As previous studies suggested, sexual dimorphism is common in *Dendrocerus* and to associate the female and male of the same species, we sequenced the gene marker 28S rDNA. DNA was extracted from a female and male of each putative species using the TIANamp Genomic DNA Kit (TIANGEN, Changping Distrit, Beijing, cat. Num. DP3400), following the protocol used by Taekul et al. (2013). The primers for 28S amplification are D2-3549F (5’-AGTCGTGTTGTGTGTGCAG-3’) and D2-4-68R (5’-TTGGTCGTTTCAAGCGGG-3’) (Zhang et al. 2008). Polymerase chain reactions (PCRs) were performed using a 25 μl system and conducted in a T100 Thermal Cycler (Bio-Rad). Thermocycling conditions were: an initial denaturing step at 94°C for 1 min, followed by 35 cycles of 94°C for 1 min, 50°C for 30 s, 72°C for 30 s and an additional extension at 72°C for 5 min. Amplicons were directly sequenced in both directions with forward and reverse primers by GENERAL BIOL (Anhui, China). Chromatograms were assembled with Sequencing Analysis 6 (Thermo Fisher Scientific, Gloucester, UK). Sequences generated from this study are deposited in GenBank (for accession numbers, see Table 2). Sequences of four *Dendrocerus* species generated from a previous study (Wang et al. 2021) were downloaded from GenBank (Table 2).

The genetic distances were calculated using the Kimura 2-parameter (K2P) model in MEGA X (Kumar et al. 2018). Sequences were aligned using MEGA X (Pais et al. 2014). The alignment was analysed using IQ-TREE (Minh et al. 2020) to generate a Maximum Likelihood (ML) tree to show the affinities amongst the studied species. Sequences of two *Ceraphron* species (accession Nos. MH733890 and GQ374733) were downloaded from GenBank and used as outgroups.

## Taxon treatments

### Dendrocerus lui

Li and Wang, 2023 sp. nov.

81E6F423-EE09-5173-A8C6-6715273D13B0

B6E23506-EF00-4B78-9377-BDE73AB6EE86

#### Materials

- **Type status:** Holotype. **Occurrence:** sex: male; lifeStage: adult; associatedSequences: GenBank OR120392; occurrenceID: B8B29425-304A-5D0F-837E-4217CAECD7A8; **Taxon:** scientificName: *Dendroceruslui*; kingdom: Animalia; phylum: Arthropoda; class: Insecta; order: Hymenoptera; family: Megaspilidae; genus: Dendrocerus; specificEpithet: *lui*; taxonRank: species; **Location:** country: China; stateProvince: Chongqing; county: China; municipality: Linkouzi; **Event:** year: 2022; month: 8; day: 22-26; verbatimEventDate: 22-26/08/2022; **Record Level:** language: en; rightsHolder: Anhui Provincial Key Laboratory of the Conservation and Exploitation of Biological Resources, Anhui Provincial Key Laboratory of Molecular Enzymology and Mechanism of Major Diseases, College of Life Sciences, Anhui Normal University; institutionCode: Anhui Normal University (AHN)
- **Type status:** Paratype. **Occurrence:** sex: 2 females; lifeStage: adult; associatedSequences: GenBank:OR120391; occurrenceID: 98EE08D6-F526-5E40-BD13-F781C5E8BC41; **Taxon:** scientificName: *Dendroceruslui*; kingdom: Animalia; phylum: Arthropoda; class: Insecta; order: Hymenoptera; family: Megaspilidae; genus: Dendrocerus; specificEpithet: *lui*; taxonRank: species; **Location:** country: China; stateProvince: Chongqing; county: China; municipality: Luomadian; verbatimCoordinates: 31°27'N, 109°56'E; verbatimLatitude: 31°27'; verbatimLongitude: 109°56′; **Event:** year: 2022; month: 8; day: 10; verbatimEventDate: 10/08/2022; **Record Level:** language: en; rightsHolder: Anhui Provincial Key Laboratory of the Conservation and Exploitation of Biological Resources, Anhui Provincial Key Laboratory of Molecular Enzymology and Mechanism of Major Diseases, College of Life Sciences, Anhui Normal University; institutionCode: Anhui Normal University (AHNU)
- **Type status:** Paratype. **Occurrence:** sex: 2 females; lifeStage: adult; occurrenceID: 9F1FF89D-7973-5B4A-B321-66E6400CA2EE; **Taxon:** scientificName: *Dendroceruslui*; kingdom: Animalia; phylum: Arthropoda; class: Insecta; order: Hymenoptera; family: Megaspilidae; genus: Dendrocerus; specificEpithet: *lui*; taxonRank: species; **Location:** country: China; stateProvince: Chongqing; county: China; municipality: Luomadian; verbatimCoordinates: 31°27'N, 109°56'E; verbatimLatitude: 31°27'; verbatimLongitude: 109°56′; **Event:** year: 2022; month: 6; day: 28; verbatimEventDate: 28/06/2022; **Record Level:** language: en; rightsHolder: Anhui Provincial Key Laboratory of the Conservation and Exploitation of Biological Resources, Anhui Provincial Key Laboratory of Molecular Enzymology and Mechanism of Major Diseases, College of Life Sciences, Anhui Normal University; institutionCode: Anhui Normal University (AHNU)

#### Description

**Male**: Body length: 1.9 mm.

**Figure 1.**
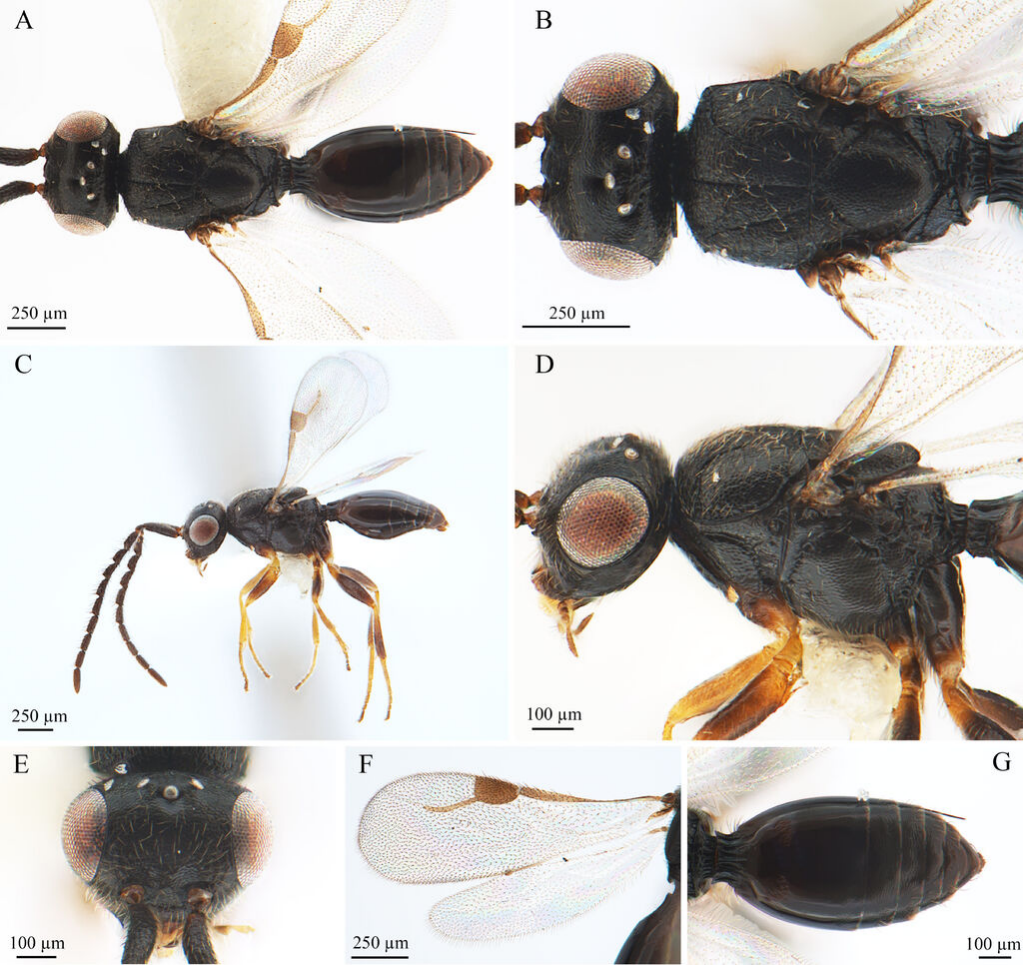
*Dendroceruslui* Li and Wang, sp. nov., male, holotype. **A** dorsal habitus; **B** head and mesosoma, dorsal view; **C** lateral habitus; **D** head and mesosoma, lateral view; **E** head, anterior view; **F** wings; **G** metasoma, dorsal view.

**Colouration** (Fig. 1): Head, mesosoma and metasoma black. Base of the pedicle segment light brown and the rest black. Mouthparts, maxilla, mandible and middle of eyes light brown; margin of eyes and ocelli slightly silvery. Legs yellowish-brown; coxa, middle of the tibia and tibial segments and ends of the tarsus dark brown to black. Transparent wing, stigma light coffee colour. Male genitalia light brown.

**Head** (Fig. 1C): Slightly wider than mesosoma (about 1.2× wider than mesosoma). HH:EHf = 2. HH:HL = 1.4. HW:IOS = 1.7. HW:HH = 1.1. POL:OOL = 1.2. Ocellar triangle with broad base, OOL:LOL = 2.2. Head shape oval in side view. Facial pit present and shallow, females not present. Preocellar pit small, ocellar fovea and present (Fig. 1B). Preoccipital lunula present, more distinct and transverse in males than in females. Preoccipital furrow present. Upper of the scrobes W shape, intertorular carina absent (Fig. 1E). Frons with sparse hairs, forehead setae free.

**Antennae** (Fig. 1C): Scape nearly three times longer than wide, pedicel small and almost a droplet. Scape length vs. pedicel length: 4. Scape length vs. F1 length: 1.8. F1 length vs. pedicel length: 2.3. F1 length vs. F2 length: 1.2. Scape is equal to the sum of lengths F1 and F2. Longest flagellomere: F9. F1-F5 trapezoidal, about 4 times as long as broad. F1-F9 pubescence gradually shortening.

**Mesosoma** (Fig. 1B, D): Mesosoma slightly narrow (1.4× longer than wide) (Length/width/height = 700/450/500 µm); densely pubescent, alutaceous in sculpture; mesoscutum: (length/width: 290/450), mesoscutum 1.6× wider than long, (Ascw/Pscw: 390/340). Shallow sulcus on the mesoscutum lying lateral to the notaulus and parallel to median mesoscutal sulcus, half the length of the mesoscutum. Scutellum: (length/width: 280/290), scutellum width almost equal to length; scutoscutellar sulcus foveolate, continuous with interaxillar sulcus. Axilla width slightly longer than length. Posterior of scutellum foveolate. Pronotum triangular with a raised area and a circular depression on the upper right. Anterior mesopleural sulcus foveolate.

**Wings** (Fig. 1F): Forewing length 1.4 mm. Hyaline, densely pubescent and marginal fringes numerous. Stigma (length/width: 190/120) 1.6× as long as wide, semicircular, posterior margin (part of pterostigma) straight. Radius (210 µm), curved a little in the latter and slightly longer (1.1×) than stigma. Hind-wing without venation.

**Metasoma** (Fig. 1G): Metasoma shaped oval, slightly elongated. Metasoma 2× longer than wide (length/width/height: 840/410/370). Metasoma smooth, but with reticular pattern on the back half of the metasoma. Syntergum reaching 56% of metasomal length. Five grooves gastral reaching 22% length of syntergum. Syntergal translucent patch present, heart-shaped. Syntergum absent.

**Figure 2.**
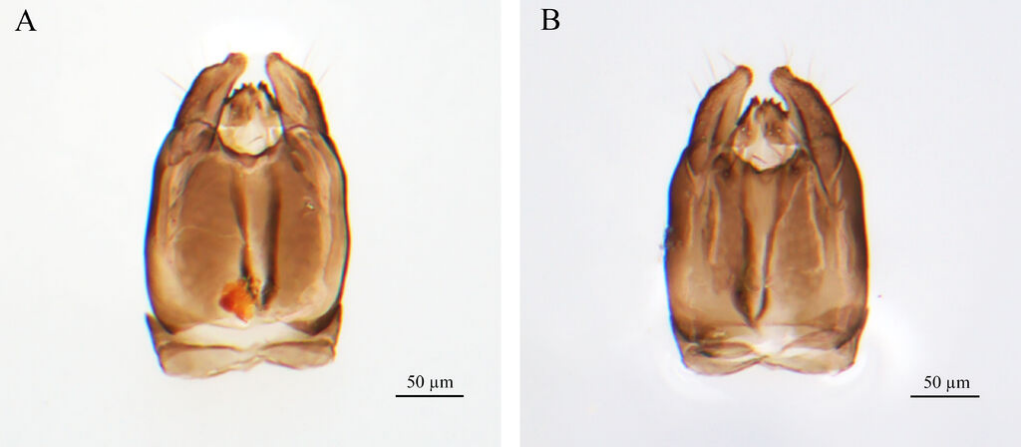
*Dendroceruslui* Li and Wang, sp. nov., male, holotype, genitalia. **A** dorsal view; **B** lateral view.

**Male genitalia** (Fig. 2): Genitalia with short cupula. Harp cylindroid (terminal constriction), shorter than gonostipes (reaching 3/5 to gonostipes), setae (part of harpe) length equal to harpe width. Gonostipes as wide as long; parossiculus fused with gonostipes. Setae (part of parossiculus) present apically. Gonossiculus with four spines apically. Penis valva terminal bending inwards.

**Figure 3.**
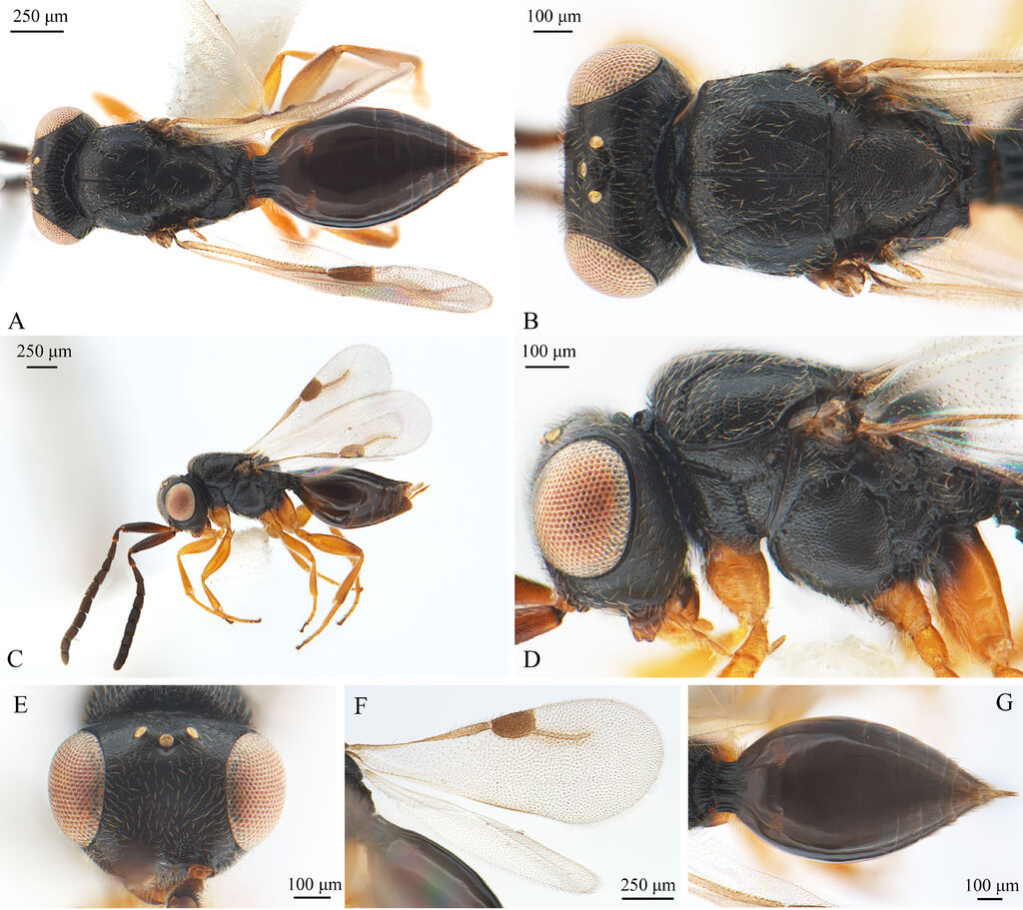
*Dendroceruslui* Li and Wang sp. nov., female, paratype. **A** dorsal habitus; **B** head and mesosoma, dorsal view; **C** lateral habitus; **D** head and mesosoma, lateral view; **E** head, anterior view; **F** wings; **G** metasoma, dorsal view.

**Females** (Fig. 3): Same as the males, except for the following characters: Body length: 2.1–2.4 mm. Antennal scape long, slightly longer than the length of flagellum 1, 2, 3 combined; flagellum gradually expanded to the end. Legs tawny, coxa blackish-brown. The metasoma fusiform.

#### Diagnosis

This new species belongs to the *D.carpenteri* species-group as indicated by the morphology of the male antennae: the outline of the flagellum is serrated at the base, but the length of the flagellum is greater than the width, with a triangular or trapezoidal outline. Then it was compared with the other 12 known species (Suppl. material 1) of this species-group. It can be classified into five types, based on the difference of antennae in *D.carpenteri* species-group: F1-F4 are trapezoidal, F1-F5 are trapezoidal, F1-F7 are trapezoidal, F1-F8 are trapezoidal and F2-F4 are trapezoidal (Suppl. material 1). Of these, F1-F5 only in *D.lui* **sp. nov.** and *D.liebscheri* are trapezoidal. However, the notauli of *D.lui* **sp. nov.** are not convergent posteriorly (the notauli of *D.liebscheri* are strongly convergent and connected posteriorly).This new species can be separated from other species by the following characters: head, mesosoma and metasoma black; five gastral carinae, syntergal translucent patch heart-shaped, harpe of male genitalia cylindroid, digitiform apically.

#### Etymology

In recognition of the collector Decai Lu, this species is named after the surname of the collector.

#### Distribution

China (Chongqing).

## Identification Keys

### Key to the species of *Dendrocerus* from China (based on males)

**Table:** 

1	Antennae ramose	2
–	Antennal flagellum triangular or trapezoidal	3
2	Ramose antenna with five branches	*D.angustus* Dessart, 1999
–	Ramose antenna with six branches	*D.anisodontus* Wang, Chen and Mikó, 2021
3	Antennal flagellum triangular	*D.bellus* Wang, Chen and Mikó, 2021
–	Antennal flagellum trapezoidal	4
4	Mesoscutum with notauli complete	5
–	Mesoscutum with notauli incomplete	6
5	Base of 3^rd^ tergite of abdomen with fine longitudinal striae; stigma of forewing narrow; coxae yellow	*D.laticeps* Hedicke, 1929
–	Base of 3^rd^ tergite of abdomen with coarse longitudinal striae; stigma wider; coxae black	*D.laevis* Ratzeburg, 1852
6	Legs all black	*D.carpenteri* Curtis, 1829
–	Legs yellowish, tibiae and femora light brown medially	7
7	Facial pit present; top of genitalia harp without contraction	*D.aphidum* Rondani, 1877
–	Facial pit present; top of genitalia harp without contraction	*D.lui* Li and Wang, **sp. nov.**

## Discussion

**Figure 4.**
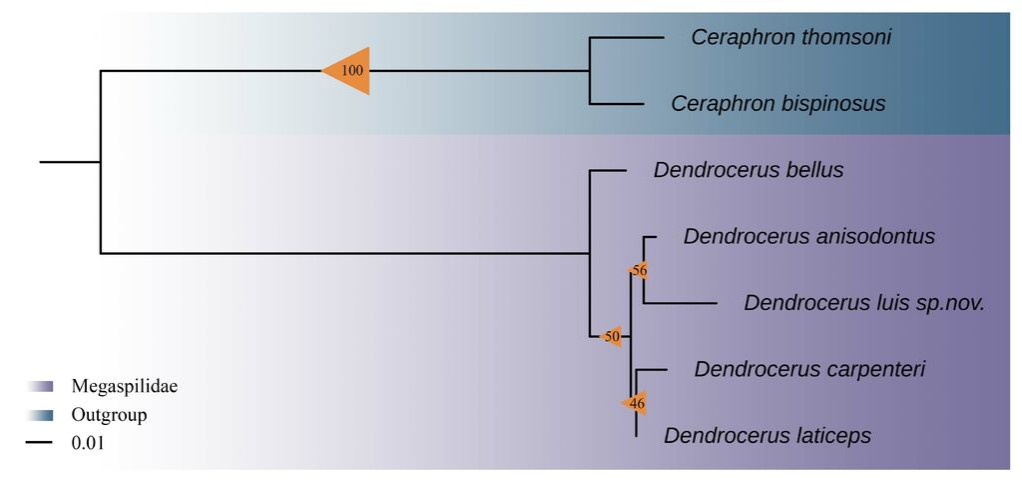
The genus *Dendrocerus* relationships based on Maximum Likelihood of 28S. The triangles of different sizes refer to level of ultrafast bootstrap support values. The scale bar represents number of substitutions per site.

In this study, we generated two 28S sequences both from the female and male of a *Dendrocerus* species, which is described as new to science below based on morphology. Sequences of both sexes of the new species are identical, confirming the correct association of female and male of the species. The genetic distances between the new species and other four species from China were 0.022 to 0.043 (Table 3). Each species recovered on the tree is clearly separated from all neighbouring species, as shown in Fig. 4.

According to the phylogenetic tree, *D.anisodontus* and *D.lui* are sister groups. However, in morphological terms, the two species appear to be very different. *D.anisodontus* has branching antennae belonging to the *D halidayi* species-group, whereas *D.lui* has trapezoidal or triangular antennal flagellae belonging to the *D.carpenteri* species-group. The head of *D.anisodontus* is rougher than that of *D.lui*. There is a large degree of structural similarity between the lateral thorax of *D.anisodontus* and that of *D.lui.* The abdomen of *D.anisodontus* has many incised points, whereas the abdomen of *D.lui* is smoother. The harpe of the male genitalia of *D.anisodontus* has more than 12 setae, while the harpe of the male genitalia of *D.lui* only has 5-6 setae. Biogeographically, *D.anisodontus* occurs in several provinces in northern, southern and eastern China, whereas *D.lui* was only collected in Chongqing Municipality in western China.

## Supplementary Material

XML Treatment for Dendrocerus lui

CCAC3DBF-D9CB-5008-A5D5-04F42C4DA5B4 10.3897/BDJ.11.e108742.suppl1 Supplementary material 1 Supplementary table 1 Data type table Brief description The 12 known species of *D.carpenteri* species-group and the characteristics of antennae. File: oo_894523.pdf https://binary.pensoft.net/file/894523 Fang Li, Shanshan Cui, Yixin Huang, Xuan Wang, Huayan Chen, Xu Wang

## Figures and Tables

**Table 1.** Abbreviations and morphological terms used in text.

**Abbreviations**	**Paraphrase**
F1, F2, ..., F9	Flagellum 1, 2, ..., F9.
LOL	Lateral ocellar length, shortest distance between inner margins of median and lateral ocelli.
OOL	Ocular ocellar length, minimum distance between a posterior ocellus to the eye margin.
POL	Posterior ocellar length, shortest distance between inner margins of posterior ocelli.
HH	Head height, lateral view.
EHf	Eye height, anterior view.
HL	Head length.
HW	Head width.
IOS	Interorbital space.
AscW	Anterior mesoscutal width.
PscW	Posterior mesoscutal width.

**Table 2.** Detailed information of sequenced samples and accession numbers.

**Species**	**Sex**	**GenBank Accession No.**	**Reference**
*D.carpenteri*	male	MZ340592	Wang et al. (2021)
	female	MZ340590	Wang et al. (2021)
*D.laticeps*	male	MZ340593	Wang et al. (2021)
	female	MZ340624	Wang et al. (2021)
*D.bellus*	male	MZ344975	Wang et al. (2021)
	female	MZ344976	Wang et al. (2021)
*D.anisodontus*	male	MZ344977	Wang et al. (2021)
	female	MZ344978	Wang et al. (2021)
*D.lui* **sp.nov.**	male	OR120392	This study
	female	OR120391	This study

**Table 3.** Genetic distance of 28S of five *Dendrocerus* species from China.

	**1**	**2**	**3**	**4**	**5**	**6**	**7**	**8**	**9**	**10**
1. *D.carpenteri*, male										
2. *D.carpenteri*, female	0.000									
3. *D.laticeps*, female	0.008	0.008								
4. *D.laticeps*, female	0.008	0.008	0.000							
5. *D.bellus*, female	0.031	0.031	0.022	0.022						
6. *D.bellus*, male	0.031	0.031	0.022	0.022	0.000					
7. *D.anisodontus*, female	0.013	0.013	0.008	0.008	0.027	0.027				
8. *D.anisodontus*, male	0.013	0.013	0.008	0.008	0.027	0.027	0.000			
9. *D.lui* sp. nov., male	0.033	0.033	0.024	0.024	0.043	0.043	0.022	0.022		
10. *D.lui* sp. nov., female	0.033	0.033	0.024	0.024	0.043	0.043	0.022	0.022	0.000	
