# Supplementary material for: Dendroceruslui (Hymenoptera, Ceraphronoidea, Megaspilidae): a new species of Dendroceruscarpenteri species-group from China
Source: Biodivers Data J. 2023 Oct 9;11:e108742. doi: 10.3897/BDJ.11.e108742 (PMC10840502; doi:10.3897/BDJ.11.e108742)
Supplement: Supplementary material 1 — Supplementary table 1 [file bdj-11-e108742-s001.pdf]

Table 1 The 12 known species of *D. carpentieri* species-group and the characteristics of antennae.

	Species	Antennae	Reference
1	*Dendrocerus bifoveatus*	F1-F4 trapezoidal	Alekseev, V.N., & Radchenko, T.D. (2001). CERAPHRONOID WASPS (HYMENOPTERA, CERAPHRONOIDEA) OF THE FAUNA OF THE UKRAINE COMMUNICATION 1.<br>Hodkinson, Ian & Buhl, Peter & Coulson, Steve & Webb, Nigel. (2003). First record of Dendrocerus bifoveatus (Kieffer, 1 907) (Hymenoptera: Ceraphronoidea) from Svalbard.
2	*Dendrocerus basalis*	F1-F4 trapezoidal	Alekseev, V.N., & Radchenko, T.D. (2001). CERAPHRONOID WASPS (HYMENOPTERA, CERAPHRONOIDEA) OF THE FAUNA OF THE UKRAINE COMMUNICATION 1.
3	*Dendrocerus remaudierei*	F1-F4 trapezoidal	Alekseev, V.N., & Radchenko, T.D. (2001). CERAPHRONOID WASPS (HYMENOPTERA, CERAPHRONOIDEA) OF THE FAUNA OF THE UKRAINE COMMUNICATION 1.
4	*Dendrocerus liebscheri*	F1-F5 trapezoidal	Alekseev, V.N., & Radchenko, T.D. (2001). CERAPHRONOID WASPS (HYMENOPTERA, CERAPHRONOIDEA) OF THE FAUNA OF THE UKRAINE COMMUNICATION 1.
5	*Dendrocerus carpenteri*	F1-F7 trapezoidal	Occurrence 1057141249 (gbif.org)<br>Isshiki, S. (1973). STUDIES ON APHID HYPERPARASITES OF JAPAN, 1 - APHID HYPERPARASITES OF THE GENUS DENDROCERUS RATZEBURG OCCURRING IN JAPAN (HYMENOPTERA : CERAPHRONIDAE) -. Insecta Matsumurana, 2, 1-37.
6	*Dendrocerus laticeps*	F1-F7 trapezoidal	Isshiki, S. (1973). STUDIES ON APHID HYPERPARASITES OF JAPAN, 1 - APHID HYPERPARASITES OF THE GENUS DENDROCERUS RATZEBURG OCCURRING IN JAPAN (HYMENOPTERA : CERAPHRONIDAE) -. Insecta Matsumurana, 2, 1-37.
7	*Dendrocerus psyllarum*	F1-F7 trapezoidal	Occurrence 3732353641 (gbif.org)<br>Isshiki, S. (1973). STUDIES ON APHID HYPERPARASITES OF JAPAN, 1 - APHID HYPERPARASITES OF THE GENUS DENDROCERUS RATZEBURG OCCURRING IN JAPAN (HYMENOPTERA : CERAPHRONIDAE) -. Insecta Matsumurana, 2, 1-37.
8	*Dendrocerus constrictus*	F1-F8 trapezoidal	Dendrocerus constrictus (Brues, 1909) (gbif.org)；<br>Isshiki, S. (1973). STUDIES ON APHID HYPERPARASITES OF JAPAN, 1 - APHID HYPERPARASITES OF THE GENUS DENDROCERUS RATZEBURG OCCURRING IN JAPAN (HYMENOPTERA : CERAPHRONIDAE) -. Insecta Matsumurana, 2, 1-37.
9	*Dendrocerus aphidum*	F1-F8 trapezoidal	Occurrence 3716302433 (gbif.org)；
10	*Dendrocerus laevis*	F1-F8 trapezoidal	Isshiki, S. (1973). STUDIES ON APHID HYPERPARASITES OF JAPAN, 1 - APHID HYPERPARASITES OF THE GENUS DENDROCERUS RATZEBURG OCCURRING IN JAPAN (HYMENOPTERA : CERAPHRONIDAE) -. Insecta Matsumurana, 2, 1-37.
11	*Dendrocerus bicolor*	F1-F8 trapezoidal	Isshiki, S. (1973). STUDIES ON APHID HYPERPARASITES OF JAPAN, 1 - APHID HYPERPARASITES OF THE GENUS DENDROCERUS RATZEBURG OCCURRING IN JAPAN (HYMENOPTERA : CERAPHRONIDAE) -. Insecta Matsumurana, 2, 1-37.
12	*Dendrocerus breadalbimensis*	F2-F4 trapezoidal	Alekseev, V.N., & Radchenko, T.D. (2001). CERAPHRONOID WASPS (HYMENOPTERA, CERAPHRONOIDEA) OF THE FAUNA OF THE UKRAINE COMMUNICATION 1.
